# FoxM1 Promotes Glioma Cells Progression by Up-Regulating Anxa1 Expression

**DOI:** 10.1371/journal.pone.0072376

**Published:** 2013-08-26

**Authors:** Shi-Xiang Cheng, Yue Tu, Sai Zhang

**Affiliations:** Institute of Traumatic Brain Injury and Nervous Diseases of Chinese People’s Armed Police Forces, Center for Neurology and Neurosurgery of Affiliated Hospital of Logistics College of CPAPF, Tianjin, China; The Ohio State University, United States of America

## Abstract

Forkhead box M1 (FoxM1) is a member of the forkhead transcription factor family and is overexpression in malignant gliomas. However, the molecular mechanisms by which FoxM1lead to glioma carcinogenesis and progression are still not well known. In the present study, we show that Anxa1 was overexpression in gliomas and predicted the poor outcome. Furthermore, Anxa1 closely related to the FoxM1 expression and was a direct transcriptional target of FoxM1. Overexpression of FoxM1 up-regulated Anxa1 expression, whereas suppression of FoxM1 expression down-regulated Anxa1 expression in glioma cells. Finally, FoxM1 enhanced the proliferation, migration, and angiogenesis in Anxa1-dependent manner both *in vitro* and *in vivo*. Our findings provide both clinical and mechanistic evidences that FoxM1 contributes to glioma development by directly up-regulating Anxa1 expression.

## Introduction

Gliomas are the most common tumors of the central nervous system in adults. The most common form of glioma in humans is astrocytoma, which, according to the World Health Organization (WHO) classification [1], comprises pilocytic astrocytoma, low-grade astrocytoma, anaplastic astrocytoma, and glioblastoma multiforme. Despite recent advances in both diagnostic modalities and therapeutic strategies, glioma remains one of the deadliest human cancers. The 5-year survival rate in patients with glioma is among the lowest for all cancers [2]. In patients with glioblastoma multiforme, the median patient survival remains about one year [3]. Advances in the treatment of malignant glioma will require an improvement in the understanding of the biology and molecular mechanisms of glioma development and progression.

FoxM1 is a member of the Forkhead box (Fox) transcription factor family, which is a predominant regulator of the cell cycle [4]–[7]. Previous studies have found that FoxM1 is predominantly expressed at the mRNA level in fetal tissue, whereas its expression is extinguished in differentiated cells [4], [5]. Furthermore, FoxM1 is substantially elevated in several aggressive human carcinomas and can contribute to oncogenesis in many tissue types, including gliomas, breast, hepatocellular, prostate, lung, and colorectal cancers [8]–[13]. Increasing evidence indicates that FoxM1 plays an important role in gliomagenesis. For example, aberrant FoxM1 expression was a common molecular alteration in malignant glioma [14], [15]. FoxM1B was the predominant FoxM1 isoform in human gliomas but not in normal brain tissue [12].

Annexin A1 (Anxa1) is a member of a family of calcium/phospholipid-binding proteins. Using proteomic and genomic analyses, Anxa1 and its member Anxa2 has been identified to be candidate regulators during carcinogenesis [16]–[18]. Recent studies indicate that Anxa1 has a wide variety of cellular functions, including membrane aggregation, phagocytosis, proliferation, apoptosis and carcinogenesis [19]. Anxa1 is overexpression in patients with hepatocellular carcinoma, gliomas, and adenocarcinomas of the esophagus and pancreas, but loss of expression in adenocarcinoma of the breast and prostate, as well as in squamous cell carcinoma of the esophagus and head and neck [20], [21]. These evidences suggest that Anxa1 has different role in different type cancers. Previous studies showed that Anxa1 is associated with metastasis in several invasive malignancies [22]–[24]. Accordingly, Anxa1 knockout mice present defects in tumor growth, metastasis, angiogenesis and wound healing suggesting the importance of Anxa1 in regulating tumor progression [25], [26].

In the present study, we show that Anxa1 expression related to FoxM1 expression in human glioma tissues and predict poor outcome. Furthermore, we found that FoxM1 promote glioma cells proliferation, migration, and angiogenesis by directly regulating Anxa1 expression.

## Materials and Methods

### Human Specimens and Cell Culture

Primary human glioma specimens were obtained from Affiliated Hospital of Logistics College of CPAPF. All tumors were from patients with a newly diagnosed glioblastoma multiforme who had received no therapy before sample collection. All glioblastoma multiforme patients underwent gross total resection of their tumors and had completed conventional external beam radiation therapy after surgery. Overall survival time of the glioblastoma multiforme patients was measured from the date of diagnosis to the date of death. This study was approved by the Institutional Review Board of the Affiliated Hospital of Logistics College of CPAPF and written consent was obtained from all participants.

The human glioma cell line Hs683, SW1088, LN-229, and U-87MG were obtained from the American Type Culture Collection. The entire cell lines were maintained in DMEM (Gibco) supplemented with 10% fetal bovine serum (FBS, Gibco) and 1% penicillin/streptomycin in a humidified atmosphere of 5% CO_2_ at 37°C.

### Reverse Transcription Quantitative Real-time PCR (RT-qPCR)

Total RNA was extracted with TRIZOL reagent according to the manufacturer’s instructions. 5 µg of total RNA was used to perform reverse transcribed by using SuperScript II and oligo dT following the manufacturer recommendations (Invitrogen). The RT-qPCR analysis was performed using the Fast SYBR Green MasterMix System (Invitrogen) according to the manufacturer’s instructions. The following primers were used: 5′-GGGCGCACGGCGGAAGATGAA-3′ (forward primer) and 5′-CCACTCTTCCAAGGGAGGGCTC-3′ (reverse primer) for human FoxM1, 5′-GGTGTGAATGAAGACTTGGCTGA-3′ (forward primer) and 5′-GTTTCATCCAGGATGGCTTGGCA-3′ (reverse primer) for human Anxa1, and 5′-TGGGGAAGGTGAAGGTCGG-3′ (forward primer) and 5′-CTGGAAGATGGTGATGGGA-3′ (reverse primer) for human glyceraldehyde-3-phosphate dehydrogenase (GAPDH).

The relative quantification was given by the CT values, determined by triplicate reactions for all of the samples for targeted gene and GAPDH. The triplicate CT values of detectable gene were averaged, and the CT value of GAPDH was subtracted to obtain ΔCT. The relative mRNA expression level of targeted genes was determined as 2^−Δ*C*^ *_T_*.

### Western Blot

Whole cell lysates were prepared from glioma cells. A quantity of 30 µg of lysates per sample was separated by SDS-PAGE using 10% polyacrylamide gels and transferred to PVDF membrane which was subsequently incubated with polyclonal rabbit antibody against human FoxM1 (Cell signaling) at a dilution of 1∶1000, anti-Anxa1 (Cell signaling) at a dilution of 1∶1000 and a second antibody (anti-rabbit IgG, Santa Cruz Biotechnology) at a dilution of 1∶2500. The same membranes were stripped and blotted with an anti-β-actin antibody (Sigma) and used as loading controls. The probe proteins were detected using the Amersham enhanced chemiluminescence system according to the instructions of the manufacturer.

### Plasmids and Stable Transfection of Glioma Cells

To generate the pcDNA3.1-FoxM1 and pcDNA3.1-Anxa1 plasmid, the full-length human FoxM1 and Anxa1 were reverse transcriptase-polymerase chain reaction (RT-PCR) using total RNA from U-87MG cell line. The primers sequences were as following: FoxM1 (forward, 5′-GGATCCATGAAAACTAGCCCCCGTCGG-3′ and reverse, 5′-CTCGAGCTACTGTAGCTCAGGAATAA-3′) and Anxa1 (forward, 5′-GGATCCATGGCAATGGTATCAGA-3′ and reverse, 5′-CTCGAGGTTTAGTTTCCTCCACA-3′). The PCR product was cloned into *Bam*H I and *Xho* I sites of the mammalian expression vector pcDNA3.1 (+) (Invitrogen). The FoxM1 and Anxa1 shRNA plasmids were purchased from Santa-Cruz biotechnology. For generation of stable cell lines expressing pcDNA-3.1-FoxM1-neo or FoxM1-shRNA-neo, cells were transfected with the corresponding plasmids using Lipofectamine 2000 reagent (Invitrogen) according to the manufacturer’s instruction. Two days after transfection, cells were trypsinized, transferred to 10 cm cell culture dishes and isolated by 1 mg/ml of neomycin (G418) for ∼2 weeks. G418 resistant colonies that stably overexpressed FoxM1 or depleted FoxM1 were picked up and identified by RT-qPCR and Western blot. For generation of double transfectant (U-87-MG-RNAi-Anxa1 and SW1088-FoxM1-RNAi), we transfected pcDNA3.1-Anxa1-hygro and Anxa1-shRNA-hygro into U-87MG-RNAi and SW1088-FoxM1 cells, respectively, to rescue the Anxa1 expression and isolated by 700 mg/ml of hygromycin for ∼2 weeks. Hygromycin resistant colonies were picked up and identified by RT-qPCR and Western blot.

### Promoter Reporters and Dual-luciferase Assay

The Anxa1 promoter (–2000∼+1) was amplified by from genomic DNA of U-87MG cells and the fragment was cloned into the Bgl II and Kpn I restriction sites in the luciferase reporter plasmids pGL3-basic vector (Promega) (pGL3-Anxa1). We generated mutant Anxa1 (pGL3-Anxa1M) by Fast Mutagenesis System (TransGen Biotech). Glioma cells were transfected with the Anxa1 promoter reporter plasmids. For luciferase assay, 5×10^4^ cells per well in 12-well plates were cultured without antibiotics overnight and then transfected with pGL3-Anxa1 or pGL3-Anxa1M and pcDNA3.1-FoxM1. After 24 hours, cells were washed with phosphate-buffered saline (PBS), subjected to lysis, and their luciferase activities measured by using a dual luciferase assay kit (Promega). The results were normalized against *Renella* luciferase. All transfections were performed in triplicate.

### Chromatin Immunoprecipitation Assay

We performed chromatin immunoprecipitation assays using the chromatin immunoprecipitation assay kit from Upstate Biotechnology. Briefly, cultured cells were crosslinked with 1% formaldehyde and resuspended in 200 µL of SDS lysis buffer [1% SDS, 10 mmol/L EDTA, 50 mmol/L Tris-HCl (pH 8.1)] and sonicated on ice to shear the DNA to 500 to 2000 bp. The chromatins were precleared by incubation with protein A-Sepharose beads for 2 h at 4°C. Anti-FoxM1 antibodies were then added, and the samples were incubated overnight at 4°C. We used immunoprecipitation with normal rabbit IgG as a negative control. Immunocomplexes were precipitated for 2 h with protein A-Sepharose beads, and DNA was recovered by means of phenolchloroform extraction. We then subjected the DNA to PCR to amplify a 225 bp region (-1809 to -1585 bp) of the Anxa1 promoter using the Primers 5′-GGCACTGGTAATAGCAGC-3′ and 5′-TGTCATCAGGTGAGAAGG-3′. The PCR products were resolved electrophoretically on a 2% agarose gel and visualized by ethidium bromide staining.

### Immunohistochemistry

Immunohistochemistry (IHC) analysis for CD31 using an ABC method. Briefly, after dewaxing and hydration, 4 µm sections from formalin fixed paraffin-embedded tissue were subjected to heat-induced epitope retrieval in 0.01 M citrate buffer (pH 6.0). Endogenous peroxidase activity was blocked in 3% hydrogen peroxide. Nonspecific binding was blocked by treatment with normal horse serum at room temperature. The slides were then incubated with primary polyclonal rabbit anti-CD31 antibody (1∶200, Abcam) at room temperature. 3, 3′-diaminobenzidine (DAB) was used for color development, and hematoxylin was used for counterstaining.

### MTT, Migration, and Endothelial Cell Tube Formation Assays

For MTT assays, 2×10^3^ cells in 200 µl culture medium were plated into a well of 96-well plates. After culturing cell for an appropriate time, 10 µl of 5 mg/ml MTT (Sigma) was added into each well and cultured for 4 h. Then, the cell culture medium was replaced by 100 µl of dimethyl sulfoxide. Thirty minutes after dimethyl sulfoxide addition, the plates were placed on a microplate autoreader (Thermo). Optical density was read at 570 nm wavelength and cell growth curves were determined according to the optical density value.

Cell migration assays were performed using 8 µm pore polycarbonate membrance Transwell chambers (Costar). In briefly, 1×10^4^ cells were cultured in the upper chamber with serum-free medium. The lower chamber contained complete medium (10% fetal bovine serum). After incubation for 12 hours, cells adherent to top surface of the membrane were removed with a cotton applicator, whereas cells migrated to bottom surface were fixed with 70% methanol and stained with crystal violet. The migrated cells on the bottom surface of the membrane were photographed and counted on an inverted microscope.

Endothelial cell tube formation assay was performed according to the manufacturer’s instructions (Invitrogen). Briefly, 250 µl growth factor-reduced Matrigel was pipetted into each well of a 24-well plate and polymerized for 30 min at 37°C. Human umbilical vein endothelial cells (HUVEC) were harvested and suspended in conditioned medium. Next, 2×10^4^ HUVECs in 300 µl conditioned medium were added to each well and incubated at 37°C, 5% CO_2_ for 20 h. We then photographed the cultures under a bright-field microscope. The quantified results were expressed in number of capillary tubes formed per square millimeter.

### Xenograft Assay

Animals are anesthetized by intraperitoneal injection with ketamine/xylazine solution (200 mg ketamine and 20 mg xylazine in 17 ml of saline) at a dosage of 0.15 mg/10 g body weight. Then, a drill hole is made in the animal’s skull by using a small hand-controlled twist drill that is 1 mm in diameter. Glioma cells (1×10^6^) were injected intracranially into nude mice (n = 5, per group). Mice were euthanized when they were moribund or on day 90 after glioma cell injection. This study was carried out in strict accordance with the recommendations in the Guide for the Care and Use of Laboratory Animals of the National Institutes of Health. The protocol was approved by the Committee on the Ethics of Animal Experiments of the the Affiliated Hospital of Logistics College of CPAPF. All surgery was performed under sodium pentobarbital anesthesia, and all efforts were made to minimize suffering.

### Statistical Analysis

The survival analysis was carried out according to the methods of Kaplan and Meier. Results of *in vitro* experiments were depicted as mean ± SD and student’s t-test (two-tailed) was used to compare values of test and control samples. We determined the significance of differences in the *in vivo* data using the Mann-Whitney U test. All calculations were performed with the SPSS for Windows statistical software package (SPSS Inc). The level of significance was set to *P*<0.05.

## Results

### Anxa1 Expression Correlates with FoxM1 Overexpression in Human Primary Glioma Specimens and Predicts Poor Outcome

**Figure 1:**
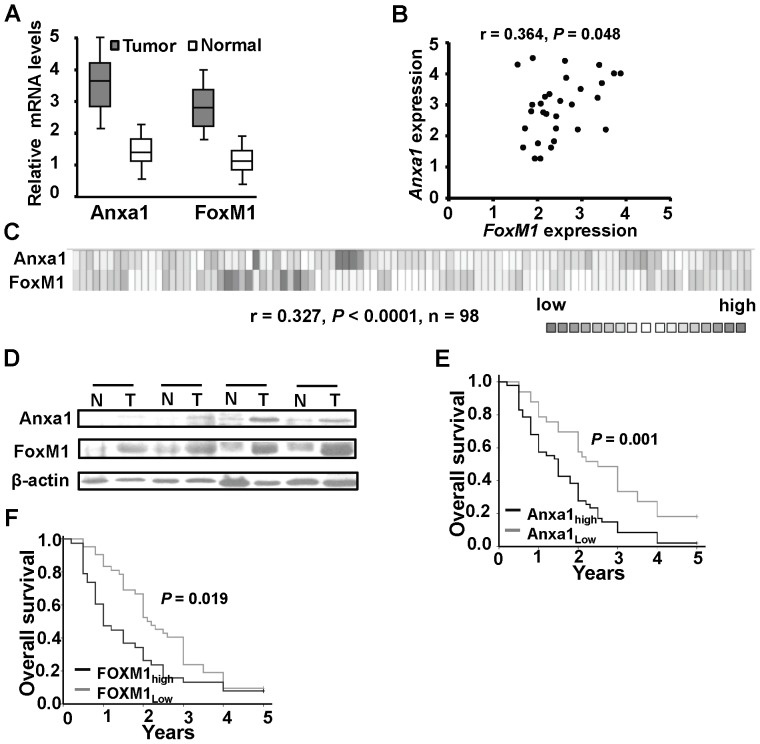
Expression of FoxM1 and Anxa1 in human normal brain and glioma tissues. **A,** Anxa1 and FoxM1 mRNA expression by RT-qPCR. The mRNA expression was analyzed in 30 matched primary glioblastoma tissues and the adjacent normal brain tissues. B, FoxM1 expression levels correlated positively with Anxa1 expression levels in glioblastoma samples (Pearson’s correlation test r = 0.364; *P* = 0.048). **C,** Headmay of a glioblastoma microarray data set from ONCOMINE data showing the expression levels of FoxM1 and Anxa1. The FoxM1 expression is correlated with Anxa1 expression (r = 0.327, *P*<0.0001). **D,** FoxM1 and Anxa1 protein expression levels in 4 matched primary glioblastoma tissues and the adjacent normal brain tissues by Western blot analysis. **E and F,** Kaplan-Meier estimates of overall survival time in patients who had a glioblastoma with different Anxa1 or FoxM1 expression.

We first determined the *Anxa1* mRNA expression in 30 human glioblastoma (grade 4) and the paired adjacent normal brain specimens by RT-qPCR analyses. The results indicated *Anxa1* mRNA expression was up-regulated in all glioma specimens (Fig. 1A). Next, we examined *FoxM1* mRNA expression in glioma specimens. Similar to the pattern we observed for Anxa1 expression, we found that *FoxM1* mRNA was also up-regulated in tumor cells than the paired adjacent normal brain tissues (Fig. 1A). In addition, we found a significant correlation between the *FoxM1* and *Anxa1* mRNA expression levels (Fig. 1B; r = 0.364, *P* = 0.048). Furthermore, we searched online ONCOMINE database (http://www.oncomine.org) to further validate our data. The publically available human glioblastoma microarray data also showed correlation between FoxM1 and Anxa1 expression (n = 98, r = 0.327, *P*<0.0001; Fig. 1C). Next, we performed Western blot analyses using total protein extracts in 4 matched human glioblastoma (T) and adjacent normal tissues (N). As shown in Fig. 1D, Anxa1 expression correlated with FoxM1 expression. The Kaplan-Meier plot of overall survival curves stratified by *Anxa1* mRNA and *FoxM1* mRNA expression was shown in Fig. 1E and 1F. Because of the distribution of *Anxa1* mRNA and *FoxM1* mRNA expression in all glioblastoma did not accord with normal distribution, ROC curve was made based on the relative level of Anxa1or FoxM1 and overall survival status of human glioblastoma patients and group the all patients into low *Anxa1* expression group (*Anxa1_low_*) and high *Anxa1* expression group (*Anxa1_high_*), and low *FoxM1* expression group (*FoxM1_low_*) and high *FoxM1* expression group (*FoxM1_high_*). Both cut-off of the Anxa1 and FoxM1were 2×10^−3^.The results indicated that both *Anxa1_high_* and *FoxM1_high_* related to poor outcome (Fig. 1E, *P* = 0.001; Fig. 1F, *P* = 0.019). Therefore, our results suggest that Anxa1 expression related to FoxM1 expression in human glioma tissues and the Anxa1/FoxM1 expression might be useful to predict the outcome in patients with glioblatoma.

### Altered FoxM1 Expression Affects Anxa1 Expression in Glioma Cells

**Figure 2:**
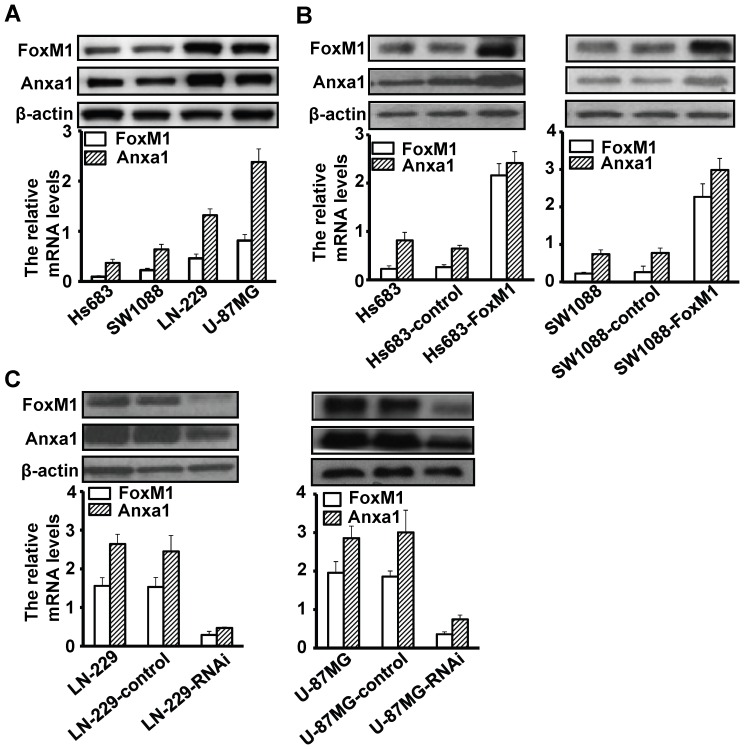
Effects of altered FoxM1 expression on Anxa1 expression in human glioma cell lines. **A,** Determination of FoxM1 and Anxa1 expression in human glioma cell lines using RT-qPCR (lower) and Western blot (upper). **B,** Up-regulation of Anxa1 mRNA and protein expression by overexpressing FoxM1. FoxM1 and Anxa1 expression levels in parental, control, and Hs683-FoxM1 and SW1088-FoxM1 cells by RT-qPCR (lower) and Western blot (upper). **C,** Down-regulation of Anxa1 mRNA and protein expression by depletion of FoxM1 expression. FoxM1 and Anxa1 expression in parental, control, and LN-229-RNAi and U-87MG-RNAi cells by RT-qPCR (lower) and Western blot (upper).

To determine the FoxM1 and Anxa1 expression levels in glioma cells lines, we examined the FoxM1 and Anxa1 expression in Hs683, SW1088, LN-229, and U-87MG cell lines by RT-qPCR and Western blot. As shown in Fig. 2A, significantly higher expression of both FoxM1 and Anxa1 were evident in LN-229and U-87MG glioblastoma cells than in Hs683 and SW1088 cells. To determine the effect of increased FoxM1 expression on Anxa1 expression, we studies two human glioma cell lines, Hs683and SW1088, that had low levels of the FoxM1 expression. We transfected these cells with FoxM1 expression vector pcDNA3.1-FoxM1 and establish two stable ectopic expression of FoxM1 clones (Hs683-FoxM1 and SW1088-FoxM1) as well as their vector control (Hs683-control and SW1088-control). We found that the FoxM1 expression was significant higher in Hs683-FoxM1 and SW1088-FoxM1 cells than the control and parental cells (Fig. 2B). These cells also exhibited significantly increased Anxa1 mRNA and protein expression (Fig. 2B). These results indicate that overexpression of FoxM1 in glioma cells increased Anxa1 expression.

Conversely, to determine the effect of decreased FoxM1 expression on Anxa1 expression, we transfected FoxM1 shRNA into LN-229 and U-87MG cells, which typically express high levels of FoxM1 and selected stable depleted FoxM1 expression clones (LN-229-RNAi and U-87MG-RNAi) as well as their vector control (LN-229-RNAi and U-87MG-RNAi). The FoxM1 mRNA and protein expression were significantly decreased in LN-229-RNAi and U-87MG-RNAi than the control and parental cells (Fig. 2C); the cells also exhibited significantly decreased Anxa1 RNA and protein expression (Fig. 2C). Our results indicate that suppression of FoxM1 expression inhibit Anxa1 expression in glioblastoma cells.

### FoxM1 Directly Binds to the *Anxa1* Promoter and Regulates it Activity in Glioma Cells

**Figure 3:**
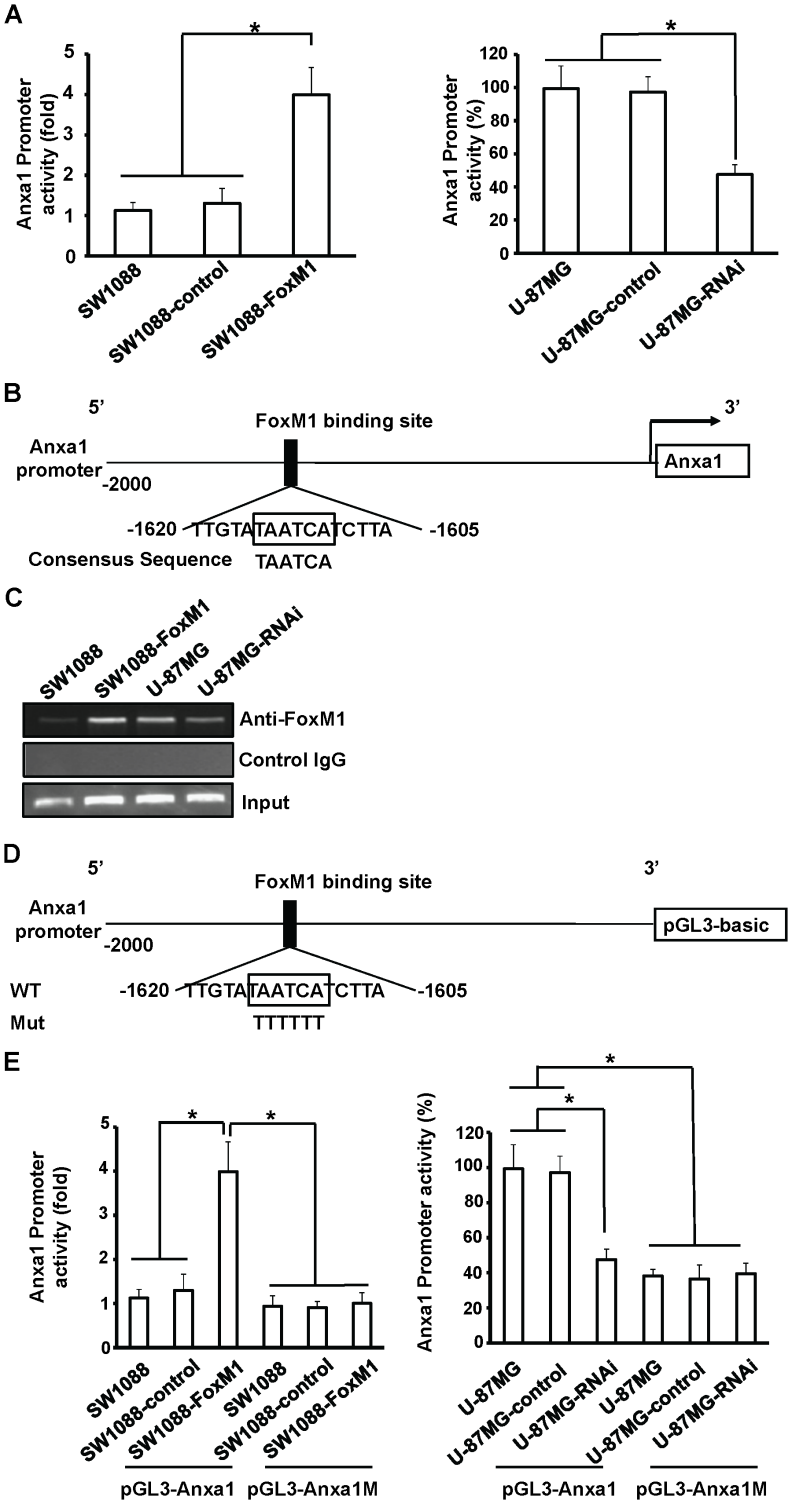
The Anxa1 as a transcriptional target of FoxM1. **A,** transactivation of the Anxa1 promoter in SW1088-FoxM1 cells (left) and repression of the Anxa1 promoter in U-87MG-RNAi cells (right). Activation was calculated relative to SW1088 cells and inhibition was calculated as a percentage relative to U-87MG cells. Three independent experiments were conducted. **B,** Sequence and position of putative FoxM1 binding site on the Anxa1 promoter. **C,** ChIP assays were done with SW1088, SW1088-FoxM1, U-87MG and U-87MG-RNAi cells. Chromatin fragments of the cells were immunoprecipitated with anti-FoxM1 antibody (top) or negative control IgG (middle) and subjected to PCR. We subjected 1% of the total cell lysates to PCR before immunoprecipitation as inputs (bottom). **D,** schematic structure of the Anxa1 promoter. The sequence of the FoxM1 binding site is shown in both wild-type (WT) and mutant (Mut) forms. **E,** Luciferase activity with or without mutation in Anxa1 promoter. SW1088-FoxM1, U-87MG-RNAi, controls and parental cells were transfected with the wild-type Anxa1 promoter or its mutant. Three independent experiments were conducted. *P<0.01.

To investigate the role of FoxM1 in regulating Anxa1 transcription, we explored whether FoxM1 regulates Anxa1 promoter activity. The Anxa1 promoter luciferase construct pGL3-Anxa1 was transfected into SW1088-FoxM1, SW-1088-control or parental cells. The luciferase activity was higher in SW1088-FoxM1 cells than the control and parental cells (Fig. 3A). Conversely, to estimate the effect of decreased FoxM1 expression on Anxa1 transcription, we transfected the pGL3-Anxa1 into the U-87MG-RNAi, U-87MG-control or parental cells. The luciferase activity was lower in U-87MG-RNAi cells than the control and parental cells (Fig. 3A). These results suggest that FoxM1 enhance the Anxa1 promoter activity in glioma cells.

To determine whether Anxa1 could be a direct transcriptional target of FoxM1, we analyzed the sequence of the Anxa1 promoter by using the FoxM1 consensus sequence 5′-TAATCA-3′ [27]. We identified a putative FoxM1 binding site in the Anxa1 promoter (Fig. 3B). To demonstrate that FoxM1 directly binds to endogenous Anxa1 promoter region, we performed chromatin immunoprecipitation assays with SW1088, SW1088-FoxM1, U-87MG and U-87MG-RNAi cells. We found that endogenous FoxM1 protein bound to the FoxM1 binding site of the Anxa1 promoter in all cell lines (Fig. 3C). Furthermore, in the SW1088-FoxM1 and U-87MG cells that expressed high FoxM1 levels, two to three times more FoxM1 protein bound to DNA promoter than in SW1088 and U-87MG cells that expressed lowFoxM1 levels (Fig. 3C). Thus, our results indicate that FoxM1 directly bind to Anxa1 promoter region *in vivo*.

To assess the functional role of the FoxM1 binding sites in Anxa1 regulation, we performed site-specific mutagenesis within the FoxM1-binding sites of the Anxa1 promoter pGL3-Anxa1. As shown in Fig. 3D, mutated Anxa1 promoter (pGL3-Anxa1M) was generated from the wild-type Anxa1 promoter construct. We transfected the pGL3-Anxa1M into SW1088-FoxM1, U-87MG-RNAi, controls and parental cells and compared the activity with that of wild-type Anxa1 promoter pGL3-Anxa1. Disruption of the FoxM1-binding site significantly attenuated Anxa1 promoter activity (Fig. 3E). These results suggest that the FoxM1 upregulates Anxa1 expression by transactivation of Anxa1 promoter in glioma cells.

### FoxM1 Enhances the Ability of Proliferation, Migration and Angiogenesis of Glioma Cells by Up-regulating Anxa1 *in vitro*

Next, we tested the function of FoxM1/Anxa1 interaction by assessing their roles in glioma cells biological behaviors. We first evaluated the ability of proliferation, migration, and angiogenesis of glioma cells, including U-87MG, LN229, SW1088, and Hs683. As described above, the expression levels of FoxM1/Anxa1 were higher in U-87MG and LN229 than SW1088 and Hs683. As shown in Fig. S1, the U-87MG and LN229 had higher proliferation, migration and angiogenesis activities than SW1088 and Hs683.

**Figure 4:**
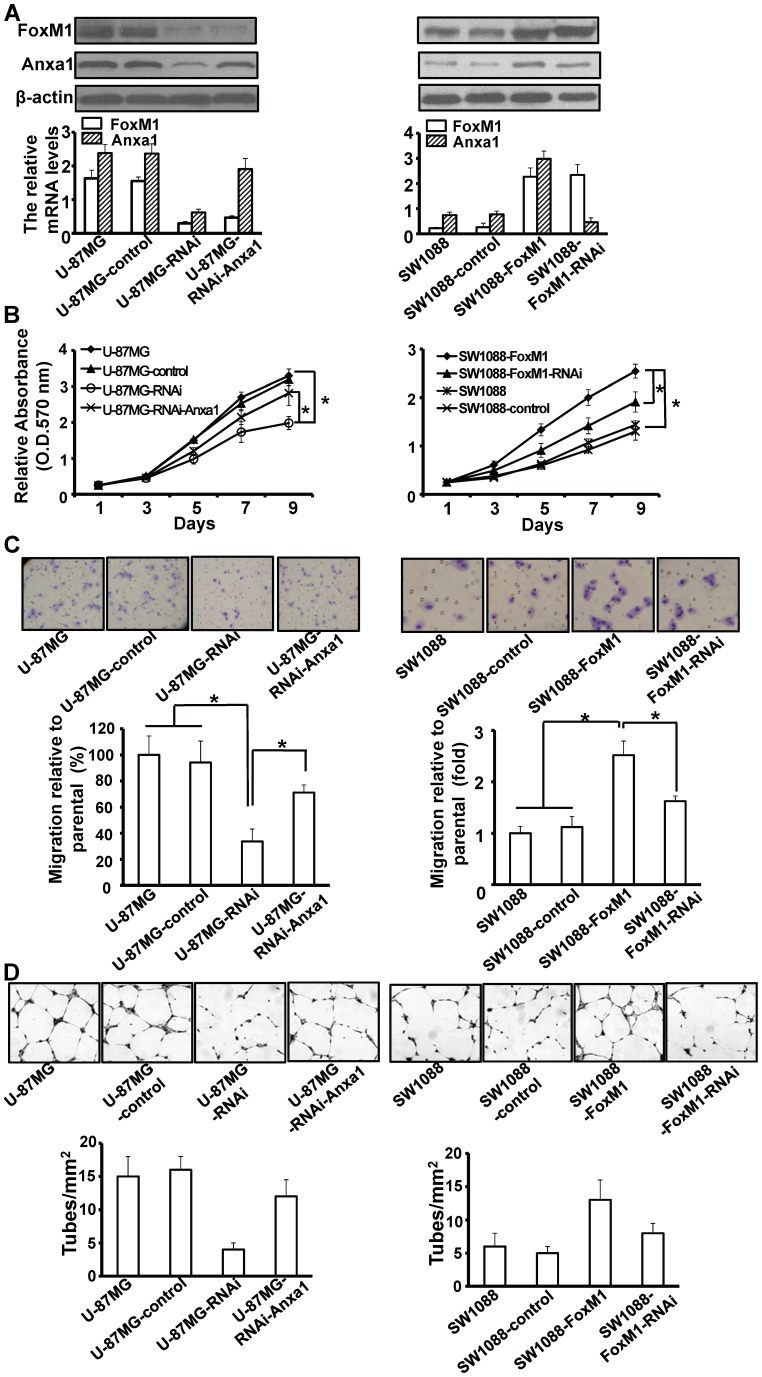
Effect of FoxM1/Anxa1 expression on proliferation and migration of glioma cells *in vitro*. **A,** RT-qPCR and Western blot analyses of FoxM1 and Anxa1 expression in stable pcDNA3.1-Anxa1-transfected U-87MG-RNAi cells (left) and Anxa1-shRNA-transfected SW0188-FoxM1 cells (right). **B,** Cells as in (A) were cultured in 96-well plates and analyzed by MTT assay. Cell proliferation curves were shown in 9 days. Three independent experiments were conducted. **C,** Cells as in (A) were examined for cell migration motility in 24-well plates with transwell chambers. Migrated cells were stained with crystal violet (upper) and counted under a light microscope (lower). Three independent experiments were conducted. **D,** the angiogenic potential of glioma cells was determined by endothelial cell tube formation assay. Capillary tube formation in each group was photographed and quantified. *P<0.05.

We next transfected pcDNA3.1-Anxa1 and Anxa1-shRNA into U-87MG-RNAi and SW1088-FoxM1 cells, respectively, to rescue the Anxa1 expression and established the stable clones (U-87MG-RNAi-Anxa1 and SW1088-FoxM1-RNAi). RT-qPCR and Western blot analyses showed that the Anxa1 mRNA and protein levels of U-87MG-RNAi and SW1088-FoxM1 were rescued in U-87MG-RNAi-Anxa1 and SW1088-FoxM1-RNAi, respectively (Fig. 4A). To observe the effects of FoxM1/Anxa1 up-regulation or down-regulation on the glioma cells, cell proliferation in stable clones overexpressing FoxM1 or depleted FoxM1 was evaluated by MTT assay. During 9-day observations, we found that the proliferation rate of SW1088-FoxM1 was apparently higher compared to the control and parental cells (Fig. 4B). Conversely, the proliferation rate of U-87MG-RNAi was significantly lower compared to the control and parental cells (Fig. 4B). Furthermore, the SW1088-FoxM1-RNAi had a lower proliferation rate than the SW1088-FoxM1 cells and the U-87MG-RNAi-Anxa1 had a higher proliferation rate than U-87MG-RNAi cells (Fig. 4B).

Furthermore, we performed transwell assays to assess the function role of FoxM1/Anxa1 in glioma cells migration. The rate of migrated cells was lower in U-87MG-RNAi cells than the control and parental cells, conversely, the migrated cells were enhanced in SW1088-FoxM1 cells compared with the control and parental cells (Fig. 4C). Moreover, FoxM1-dependent stimulation of cell migration was rescued by overexpressing or underexpressing Anxa1 in U-87MG-RNAi or SW1088-FoxM1 cells, respectively (Fig. 4C). These results indicate that FoxM1 enhances the ability of glioma cells proliferation and migration by up-regulating Anxa1 expression *in vitro*.

To provide direct evidence that FoxM1 affect the angiogenic phenotype by regulating Anxa1 of glioma cells, we determined the angiogenic ability of the U-87MG-RNAi-Anxa1 and SW1088-FoxM1-RNAi cells using an endothelial cell tube formation assay. The results indicated that the conditioned medium from U-87MG-RNAi-Anxa1 cultures had greater angiogenic potential than the conditioned medium form U-87MG-RNAi cells (Fig. 4D). In contrast, the conditioned medium from SW1088-FoxM1-RNAi cells had a reduced capillary tube formation compared with the conditioned medium from SW1088-FoxM1 cells (Fig. 4D). Together, our results indicate that FoxM1 enhances the angiogenic ability of glioma cells by up-regulating the Anxa1 expression.

### Anxa1 Overexpression Rescues the Growth of FoxM1 Down-regulated Glioma Cells in vivo

**Figure 5:**
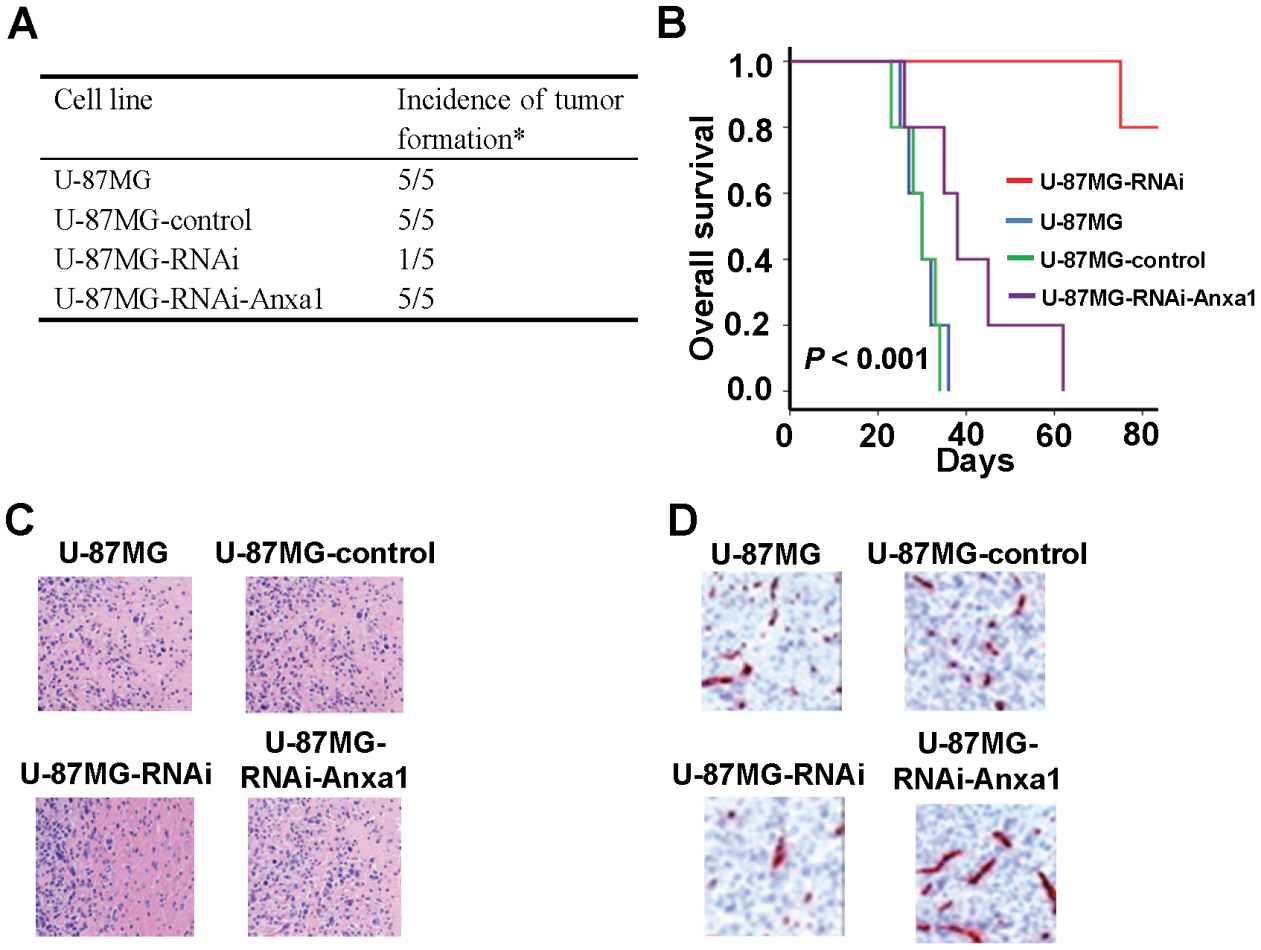
Effect of FoxM1/Anxa1 expression on glioma growth in the brains of nude mice. **A,** Glioma cells (1×10^6^) were implanted intracranially into nude mice. Mice were euthanized when they were moribund or on day 90. * Incidence: number of mice with tumor/number of mice injected. **B,** Kaplan-Meier estimates of overall survival time in nude injected with glioma cells (P<0.001). **C,** Morphologic alteration of the xenograft tumors was analyzed by H&E. D, CD31expression level in xenograft tumors was analyzed by IHC.

All the results described above pointed out a possibility that FoxM1 promote glioma progression by regulating Anxa1. To test this possibility, we intracranially injected U-87MG, U-87MG-control, U-87MG-RNAi, and U-87MG-RNAi-Anxa1 cells into nude mice and found that U-87MG, U-87MG-control, and U-87MG-RNAi-Anxa1 cells produced brain tumors in all of the injected mice, however, the U-87MG-RNAi cells produced brain tumor in only one injected mice (Fig. 5A). Furthermore, the mice became moribund ∼40 days after the injection. In contrast, the mice injected U-87MG-RNAi cells have a significant increase in overall survival time (Fig. 5B; *P*<0.001). Tissue sections H&E staining showed that the xenograft tumor formed by U-87-RNAi cells was less invasive (Figure 5C). Furthermore, xenograft tumor formed by U-87-RNAi has lower CD31 expression level. Our results indicate that inhibition of FoxM1 expression significantly suppresses the tumorigenicity of human glioblastoma cells, whereas rescue the Anxa1 expression can recover the ability of tumorigenicity.

## Discussion

In the present study, we identified both FoxM1 and Anxa1 are overexpression in the primary glioma specimens, and predicts poor survival. In addition, a strong correlation of the co-expression of FoxM1 and Anxa1 were observed in patients with gliomas. Moreover, we showed that FoxM1 promotes Anxa1 transcription in human glioma cells and Anxa1 is required for FoxM1-induced cell proliferation, migration and angiogenesis. Finally, we provided evidence that Anxa1 is required for FoxM1-induced gliomas progression in model mice. Therefore, FoxM1 overexpression contributes directly to Anxa1 overexpression in glioma cell proliferation, migration and angiogenesis.

Several studies have demonstrated the importance of FoxM1 in oncogenesis in a variety of malignancies, including gliomas [8]–[12]. Consistent with previous studies, we found that mRNA and protein levels of FoxM1 and Anxa1 were up-regulated in human glioma specimens and related to the poor outcome [10], [21], [28]. Furthermore, we found that the patients with Anxa1_high_ expression had poor outcome than the Anxa1_low_ group. These results suggested that Anxa1 expression might be useful to predict the outcome in patients with glioblatoma.

Our current study enriched the understanding of the mechanism of the FoxM1 on glioma carcinogenesis. We observed a significant correlation between FoxM1 and Anxa1 expression in glioma cells and provide evidence that FoxM1 promote glioma proliferation, migration, and angiogenesis by up-regulating Anxa1 expression. To our knowledge, this is the first report to show that Anxa1 is a direct target of FoxM1. In addition, we identified FoxM1 binding site, mapped between –1602 to –1605 of the Anxa1 promoter region. Anxa1 expression seemed to be crucially regulated by FoxM1 through direct interaction with Anxa1 promoter, as mutation of the FoxM1 binding site significantly reduced Anxa1 promoter activity in glioma cells. Furthermore, the FoxM1 expression affected the tumorigenic ability of glioma cells *in vitro* and *in vivo*, including proliferation, migration and angiogenesis, which consistent with other studies [28]–[30]. Therefore, our study provides both clinical and mechanistic evidences that FoxM1 directly regulates Anxa1 expression and showed a novel mechanism by which FoxM1 promotes glioma proliferation, migration and angiogenesis.

Recently, Schittenhelm and colleagues revealed that up-regulation of Anxa1 have a role in the development and/or progression of astrocytomas, and the highest expression were found in WHO grade IV glioblastoma [21]. Consistent with their studies, we showed that Anxa1 mRNA and protein expressions were up-regulated in glioma specimens and related to the poor outcome. Moreover, overexpression of Anxa1 in depleted FoxM1 glioma cells recovered the ability of tumorigenicity. This suggests that Anxa1 might be involved in the glioma progression, although the full functional role therein remains unclear. However, in some tumors, Anxa1 is considered to be a tumor suppressor [31]. This is possibly due to regulation of the ERK/MAPK, as Anxa1 suppresses cell proliferation by ERK mediated disruption of the actin cytoskeleton and by depletion of cyclin D1 expression [32]. Taken together, the results indicated that Anxa1 expression is highly tissue specific in different cancers. Thus, Anxa1 expression needs to be determined for each tumor type.

The mechanisms that underlie the role of Anxa1 in regulating angiogenesis are still ill defined. Most significantly, the anti-angiogenic gene TIMP2 was up-regulated and the pro-angiogenic gene SphK1 was down-regulated in Anxa1 KO experiments, whereas other important angiogenesis genes such as VEGF, FDF, Wnt remained unaffected [25]. The FoxM1-Anxa1-TIMP2-SphK1 pathway may shed light on the overall mechanisms that govern pathological versus physiological angiogenesis.

In conclusion, we found that FoxM1 directly regulated Anxa1 expression to promote glioma cells proliferation, migration, and angiogenesis. Because of the diverse function of FoxM1 in glioma tumorigenesis and progression, a better understanding of FoxM1 signaling and function will help identify effective targets for gliomas therapy.

## Supporting Information

Figure S1 The ability of proliferation, migration, and angiogenesis of glioma cells. **A,** Cells were cultured in 96-well plates and analyzed by MTT assay. Cell proliferation curves were shown in 9 days. **B,** Cells were examined for cell migration motility in 24-well plates with transwell chambers. Migrated cells were stained with crystal violet. **C,** the angiogenic potential of glioma cells was determined by endothelial cell tube formation assay.
(TIFF)
