# Supplementary figures and images for: FoxM1 Promotes Glioma Cells Progression by Up-Regulating Anxa1 Expression
Source: PLoS One. 2013 Aug 26;8(8):e72376. doi: 10.1371/journal.pone.0072376 (PMC3753245; doi:10.1371/journal.pone.0072376)

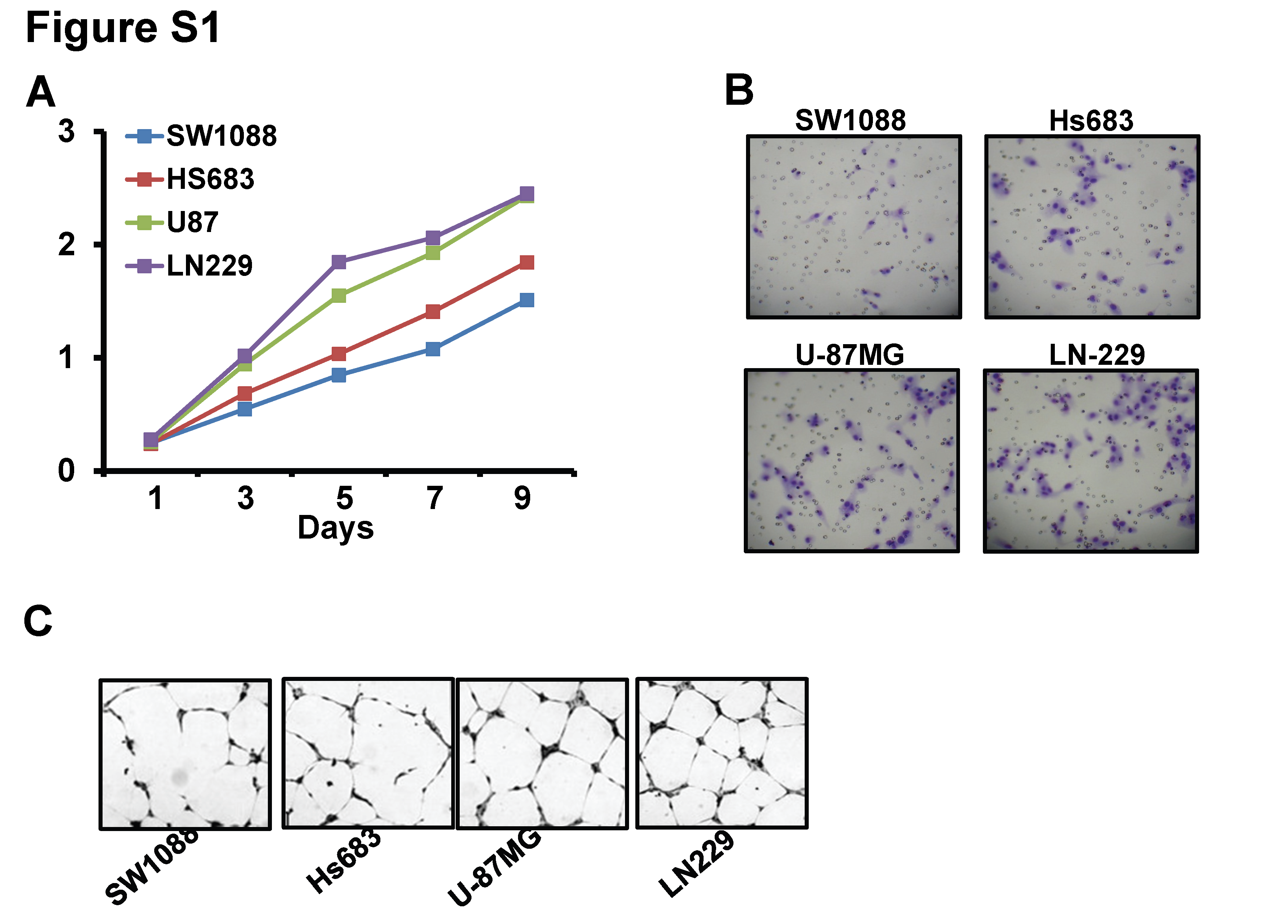

Supplement: Figure S1 — The ability of proliferation, migration, and angiogenesis of glioma cells. A, Cells were cultured in 96-well plates and analyzed by MTT assay. Cell proliferation curves were shown in 9 days. B, Cells were examined for cell migration motility in 24-well plates with transwell chambers. Migrated cells were stained with crystal violet. C, the angiogenic potential of glioma cells was determined by endothelial cell tube formation assay. (TIFF) [file pone.0072376.s001.tiff]
